# Prenatal Maternal Stress and Weak Handedness in Early Childhood: The Iowa Flood Study

**DOI:** 10.1002/dev.70143

**Published:** 2026-03-17

**Authors:** Jad Hamaoui, Hao Zhang, Suzanne King, Natalie Castellanos‐Ryan

**Affiliations:** ^1^ University Hospital Center Sainte‐Justine Montreal Quebec Canada; ^2^ Department of Psychoeducation University of Montreal Montreal Quebec Canada; ^3^ Douglas Institute Research Center Montréal Québec Canada; ^4^ Department of Psychiatry McGill University Montréal Québec Canada

**Keywords:** ambidexterity, birth‐related stressors, handedness, Iowa flood study, longitudinal study, mixed‐handedness, prenatal maternal stress

## Abstract

Weak handedness is frequently observed in individuals with neurodevelopmental and psychiatric disorders. Although birth‐related stressors, such as prematurity, have been shown to contribute to this association, the influence of early prenatal maternal stress (PNMS) remains under‐explored. This study examined the trimester‐specific effects of disaster‐related PNMS on handedness development using rare longitudinal data from the 2008 Iowa Flood Study. Pregnant women exposed to major flooding were assessed for objective (hardship severity) and subjective (psychological distress) PNMS and postnatal maternal depression, and their children's handedness was reported at 60 months (*n* = 217, 45.6% female). Path analyses revealed distinct trimester‐dependent associations between PNMS and offspring handedness. When stress exposure occurred during the first trimester, weak handedness in offspring was predicted by the child's sex, with male sex being associated with higher weak handedness (*β* = −0.29) only. In contrast, for third trimester exposure, higher objective PNMS was associated with higher weak handedness (*β* = −0.34), while the child's sex was no longer a significant predictor (*β* = 0.11). These findings suggest that PNMS influences the ontogenesis of handedness through trimester‐specific mechanisms, potentially mediated by epigenetic modifications. Potential neurobiological mechanisms underlying these associations and their clinical implications for neurodevelopmental outcomes are discussed.

## 1 Introduction

Handedness, particularly hand preference, is among the most extensively studied behavioral asymmetries in humans (Ocklenburg and Güntürkün 2024). Hand preference, defined as the preference for using one hand over the other, can be observed very early in life and emerges gradually across infancy and early childhood (Michel 2021). Behavioral asymmetries, such as prenatal thumb sucking, favoring the right or left side, can be detected as early as fetal life (Ferre et al. 2020; Hepper 2013; Hepper et al. 1991). Although there is no clear consensus regarding developmental milestones for handedness (Scharoun and Bryden 2014), postnatal preferences are evident shortly after birth. For instance, neonates more frequently show a right‐hand preference for the grasp reflex (Tan and Tan 1999) and a rightward head‐turning bias (Michel and Goodwin 1979; Rönnqvist and Hopkins 1998). Across infancy, hand preference becomes increasingly consistent, with longitudinal work suggesting that the prevalence of right‐handedness can reach approximately 76% by two years of age (Nelson et al. 2013).

Handedness, which is predominantly right‐biased in the general population (Papadatou‐Pastou et al. 2020), can be conceptualized both in terms of direction (right vs. left) and degree, reflecting the consistency of hand preference (strong vs. weak). In the present study, the degree of hand preference, rather than its direction, was adopted as the primary variable of interest. Inconsistent hand preference reflects the variability in favoring one hand over the other and can be further distinguished into two distinct types. Mixed‐handedness refers to the tendency to use one hand for some tasks and the opposite hand for others (Fagard et al. 2015). In contrast, ambidexterity is characterized by equal proficiency in using either hand for the same task (Fagard et al. 2015). Sex differences in mixed‐handedness have been observed, with males being more likely to exhibit mixed‐handedness than females (see Papadatou‐Pastou et al. 2008 for meta‐analysis).

Weak handedness, encompassing mixed‐handedness and ambidexterity, has been repeatedly associated with an increased likelihood of neurodevelopmental and psychiatric disorders (Ocklenburg et al. 2024; Packheiser et al. 2025). Several meta‐analyses have reported associations between weak handedness and developmental dyslexia (Packheiser et al. 2023), autism spectrum disorder (Markou et al. 2017), attention‐deficit/hyperactivity disorder (Nastou et al. 2022), as well as to schizophrenia (Hirnstein and Hugdahl 2014), schizotypy (Somers et al. 2009), and post‐traumatic stress disorder (Borawski et al. 2023). Recent work has shown that weak‐handedness possesses distinct characteristics compared to other handedness categories and exhibits partially unique patterns of association across psychopathology. These findings suggest that weak‐handedness may emerge from specific ontogenetic and developmental mechanisms (Packheiser et al. 2025).

Two non‐mutually exclusive theoretical hypotheses may explain the link between weak handedness and psychopathology. First, weak handedness is thought to reflect weak cerebral lateralization (Mundorf et al. 2024), a trait observed in several disorders (Mundorf et al. 2021). In this case, the association could be direct, with an early‐reduced cerebral lateralization contributing to the development of psychopathology (Bishop 2013; Hamaoui et al. 2024; Ocklenburg and Mundorf 2024). Alternatively, the association may be indirect, driven by shared genetic and non‐genetic factors that influence both reduced lateralization and psychopathology (Bishop 2013; Hamaoui et al. 2024; Ocklenburg and Mundorf 2024). Among the non‐genetic factors, stress has been proposed as a potential contributing factor (Ocklenburg et al. 2016). Early life stress is thought to disrupt typical patterns of cerebral lateralization, potentially contributing to the development of weak handedness while also increasing the risk for neurodevelopment and psychiatric disorders (Berretz et al. 2020; Hamaoui et al. 2024). Several studies have examined the relationship between birth‐related stressors and handedness, showing that risk factors such as prematurity are associated with an increase likelihood of left handedness and/or mixed handedness (Hamaoui et al. 2023; Marlow et al. 2019; see for a meta‐analysis Domellöf et al. 2011). However, stress exposure may occur during the prenatal period prior to birth, with prenatal stress arising from maternal hardships experienced during pregnancy influencing the fetal environment and subsequent development.

Previous studies have found an association between prenatal maternal stress (PNMS) and handedness in offspring, with evidence of this behavioral asymmetry emerging as early as the fetal period. For instance, maternal stress levels reported during the four weeks preceding a scan were positively associated with fetal left‐handed self‐touch between 24 and 36 weeks of gestation (Reissland et al. 2015). Regarding postnatal handedness, psychological distress—including maternal depressive symptoms, maternal anxiety, and stressful life events, such as divorce or the death of a relative—have been linked to higher rates of mixed‐handedness in exposed children at ages three (Obel et al. 2003), three and a half (Glover et al. 2004), five (Rodriguez and Waldenström 2008), and six (Gutteling et al. 2007). However, the timing of the PNMS exposure varied across studies. Rodriguez and Waldenström (2008) reported an association when PNMS was assessed at Gestational Week 16, consistent with Glover et al. (2004), who observed an association at Week 18 but not later in gestation. In contrast, Gutteling et al. (2007) and Obel et al. (2003) found stronger associations when PNMS was measured later in pregnancy, particularly during the third trimester.

These studies also differ substantially in how weak‐handedness was conceptualized and operationalized. For instance, Obel et al. (2003) classified children as mixed‐handed if they used the opposite hand for at least one activity, provided that mothers reported a consistent right‐ or left‐hand preference across all items of the questionnaire. Gutteling et al. (2007) applied a comparable criterion while excluding activities performed simultaneously with both hands. In contrast, Glover et al. (2004) operationalized mixed‐handedness using both continuous and categorical approaches, scoring the number of tasks for which children used either hand and defining mixed‐handedness as an “either‐hand” preference on two or more items, with alternative analyses using more conservative cut‐offs. Although labeled mixed‐handedness, this approach primarily captures inconsistency or lack of a dominant hand across tasks, aligning more closely with contemporary definitions of ambidexterity than with mixed‐handedness per se, which is typically defined as differential hand use across specific activities.

Taken together, substantial heterogeneity exists in how weak‐handedness has been conceptualized and operationalized across studies, which likely contributes, at least in part, to the variability in reported findings. This lack of standardization reflects the previous absence of a theoretically grounded and empirically validated framework for defining and measuring weak handedness. Consequently, in light of advances in the literature over recent decades, contemporary studies may benefit from adopting more conceptually precise and theoretically informed definitions of handedness, particularly with respect to weak handedness.

### 1.1 Present Study

Although existing research supports the association between PNMS and an increased likelihood of weak handedness in offsprings, several limitations remain, particularly in light of recent advancements in the field. The present study aims to address these gaps through three key objectives, while examining PNMS in the context of a natural disaster. This approach provides a distinct methodological advantage by providing rare data, as the exogenous, acute, and time‐limited nature of the stressor offers a unique opportunity to investigate stress exposure under real‐world conditions, that is, independent of parental characteristics, in a natural experiment.

First, prior studies have focused exclusively on mixed‐handedness or ambidexterity as indicators of weak handedness, yet these constructs have not been examined simultaneously within the same study. Moreover, recent research has introduced refined criteria for classifying mixed‐handedness and ambidexterity (Mundorf et al. 2024). The present study considers both mixed‐handedness and ambidexterity and applies updated classification criteria to assess weak‐handedness in children more precisely. Second, it is unclear whether the observed associations between PNMS and weak handedness are driven primarily by the objective hardship experienced during the stressor, by the mother's subjective distress, or both. To disentangle these effects, we examine the relative contributions of objective hardship due to a natural disaster and subjective maternal distress in predicting weak handedness in children. Third, given the inconsistencies in prior research regarding the timing of PNMS exposure and its effects on handedness development, we compare different gestational time points of exposure to identify sensitive periods when PNMS may exert the strongest influence. Determining these critical windows could offer deeper insights into the neurodevelopmental mechanisms underlying cerebral and behavioral lateralization.

We hypothesize that both higher objective and subjective PNMS will be associated with an increased weak handedness in offspring. Specifically, exposure to PNMS during the second and third trimesters of pregnancy is expected to be particularly linked to increased weak handedness. This hypothesis is grounded in the premise that PNMS during this critical developmental period may disrupt the maturation of the motor system and the development of hand asymmetries, which can emerge as early as Gestational Week 18 (Parma et al. 2017).

## 2 Method

### 2.1 Participants

The June 2008 flooding in the Midwest United States had been ranked as the worst ever disaster in the state of Iowa to date, with total damages reaching nearly $10 billion (USDOC 2009). The Iowa Flood Study, a longitudinal cohort study, followed pregnant women exposed to this natural disaster to investigate the effects of disaster‐related PNMS on a range of child behavioral, cognitive, and physical outcomes. Participant recruitment began one month after the flood's peak on June 15, 2008. Maternal reports provided data for 217 toddlers (99 females) assessed at 60 months.

Pregnant women, primarily from urban and suburban environments residing in three severely affected counties in Iowa (Johnson, Linn, and Black Hawk), were enrolled in the study. Approximately half of the sample had already been participating in a University of Iowa study on pregnancy‐related psychosocial stress and obstetric outcomes (Nylen et al. 2013). At the time of childbirth, the mothers had a mean age of 30.0 years (SD = 5.0). Most were married or living with a partner (95%) and were employed (76.5%). The vast majority were native English speakers (97.3%). In terms of community of descent, the sample was predominantly White (94%), with smaller proportions identifying as Asian or Pacific Islander (2.6%), Black or of African descent (2.0%), Native American (0.7%), or Hispanic (0.7%). Regarding educational attainment, 35.8% of mothers held a bachelor's degree, 23.0% a master's degree, and 8.1% a doctoral degree. Sixty‐six percent of families reported annual household incomes of $50,000 or more. Based on the Hollingshead Index of socioeconomic status (SES), which considers parental education and occupation, 93% of families were classified as Upper Middle Class or Upper Class.

The study adheres to the ethical standards outlined in the National Institutes of Health guidelines for human experimentation and the Helsinki Declaration of 1975 (revised 2008), and has received approval from the University of Iowa's Institutional Review Board. Informed consent was obtained from parents and children (through assent).

### 2.2 Measures

#### 2.2.1 Child Weak Handedness

Mothers completed a brief questionnaire to indicate their child's dominant hand at 60 months (left, right, both hands, or not sure) for five tasks: Drawing or coloring, throwing a ball, hitting things, stacking blocks, and using a spoon. This measure was modified from item sets used in prior studies examining associations between early‐life stress and handedness in children across a broad developmental range, from early to late childhood (e.g., Glover et al. 2004; Rodriguez and Waldenström 2008; Hamaoui et al. 2023). With the exception of the stacking blocks item, the activities closely overlap with those previously employed. The internal consistency of the questionnaire was very high in the present sample (Cronbach's *α* = 0.94), supporting its reliability. “Not sure” responses frequently co‐occurred with “both hands” responses and were, therefore, classified as “both hands” in the analysis.

Ambidexterity is rare (approximately 1%; Mundorf et al. 2024), and given our sample size, it was not feasible to analyze ambidexterity separately from mixed‐handedness. Consequently, mixed‐handedness and ambidexterity were combined into a single measure of weak hand preference. Several approaches have been proposed to quantify weak handedness. For mixed‐handedness, a common method is to calculate a laterality index using the formula [(R − L) / (R + L)] × 100, where R and L represent the total number of right‐ and left‐hand preferences, respectively (Mundorf et al. 2024; Vingerhoets et al. 2023). Ambidexterity could be assessed using a formula that quantify the proportion of items for which both hands are used ((A / N) × 100; Ocklenburg et al. 2015). However, the Laterality Indices Consensus Initiative (LICI; Vingerhoets et al. 2023) emphasizes that ambidexterity is ideally determined using hand performance measures rather than preference questionnaires.

Because performance‐based measures were not available in this longitudinal cohort, and to align as closely as possible with the approaches described above, we used the following laterality quotient (LQ) to classify weak hand preference:

$$\mathrm{LQ}=\left|\left[\left(R-L\right)/\left(\mathrm{total}\;\mathrm{number}\;\mathrm{of}\;\mathrm{responses}\right)\right]\times 100\right|$$

This formula, previously used by Fagard et al. (2015), offers the advantage of accounting for all response types (R, L, and “either” reponses). Notably, this LQ was used in a study of 9‐year‐old children (Hamaoui et al. 2023), where it effectively identified weak‐handed children who exhibited near‐equal performance with both hands on the pegboard test, a standardized measure of hand performance. Accordingly, in the present study, low LQ scores are interpreted as indicative of weak hand preference, with the inference that these children may exhibit relatively balanced hand performance. Since the present study focuses on consistency rather than directionality, the absolute value was applied to the quotient. As a result, the LQ scores ranged from 0 to 100, with higher scores (maximum = 100) indicate consistent right‐ or left‐handedness, whereas lower scores (minimum = 0) reflect inconsistent hand preference, capturing both mixed‐handedness (right‐ and left‐hand preferences across tasks) and ambidexterity (absence of preference for either hand). Hand preference was treated as a continuous variable. A small proportion of children (1.9%) had an LQ of 0, indicating a completely non‐lateralized hand preference. The proportion of children exhibiting hand preference on individual items increased gradually, with 2.6% demonstrating a preference on one item, 3.9% on two items, 13.6% on three items, and 20.1% on four items. The majority of children (57.8%) showed an LQ of 100, reflecting strong lateralization with exclusive use of a single hand across all assessed items.

#### 2.2.2 Prenatal Maternal Stress

**Objective hardship**: Objective hardship was measured using a tailor‐made self‐report questionnaire (The Iowa Flood 100; see Yong Ping et al. 2015 for all items and scoring) assessing four aspects of disaster‐related hardship: threat (e.g., “*Were you physically hurt*”), loss (e.g., *“Was you home damaged”*), scope (e.g., *“Days people were evacuated from home”*), and change (e.g., *“Did your family stay together”*). The Iowa Flood 100 (IF100) was based on a similar objective hardship scale developed for Project Ice Storm (Laplante et al. 2007), which has been shown to predict cognitive development (Laplante et al. 2008), immune function (Veru et al. 2014), and DNA methylation profiles (Cao‐Lei et al. 2014) of children exposed in utero to the 1998 Quebec ice storm. IF100 scores in the current sample ranged from 0 to 50, with higher scores indicating a greater degree of objective hardship.

**Subjective distress**: In order to reduce the number of variables in analyses, we created a composite measure of subjective distress from three different questionnaires, reflecting the women's psychological reaction to the flooding (i.e., composite score for mothers’ subjective stress: COSMOSS), using an approach used in previous studies (Lapierre et al. 2024). The 22‐item Impact of Event Scale‐Revised was used to assess the women's current level of post‐traumatic stress symptoms relative to a particular event (in this case, the 2008 Iowa floods), in three categories of symptoms: Intrusive thoughts and images, avoidance, and hyperarousal (Weiss and Marmar 1997). The 13‐item self‐reported Peritraumatic Distress Inventory examines the levels of distress during and immediately after the crisis as recalled at recruitment (Brunet et al. 2001). Similarly, the Peritraumatic Dissociative Experiences Questionnaire asks about dissociative experiences at the time of the crisis (Marmar et al. 1997). The total scores from these instruments were included in the composite score. Principal component analysis using data from all Iowa Flood Study participants resulted in a single factor that accounted for 76.36% of the overall variance in scores across the three original questionnaire measures. An algorithm was then derived to create the COSMOSS variable, using the following equation: COSMOSS = 0.380 (standardized Impact of Event Scale‐Revised) + 0.388 (standardized Peritraumatic Distress Inventory) + 0.376 (standardized Peritraumatic Dissociative Experiences Questionnaire).

**Timing of in‐utero exposure**: The timing of prenatal exposure to the floods was determined by calculating the number of days between the estimated conception date (derived from the medical records based on the last menstrual period or the infant's gestational age at birth) and the peak of the flood (June 15, 2008). This continuous measure was then categorized into three exposure groups corresponding to the first (Weeks 1–12; *n* = 66), second (Weeks 13–26; *n* = 88), and third (Weeks 27 to birth; *n* = 63) trimesters of gestation.

#### 2.2.3 Covariates

Maternal medical and obstetric history was obtained from hospital records. To control for key confounders, pregnancy‐related factors previously linked to handedness were considered, specifically gestational age at birth (e.g., Domellöf et al. 2011; Hamaoui et al. 2023), and birthweight (De Kovel et al. 2019). Both variables were initially treated as continuous. However, given their strong correlation (*r* = 0.57, *p* < 0.001), only gestational age was retained as a covariate to minimize multicollinearity. Postnatal maternal depressive symptoms were assessed six months postpartum using the Edinburgh postnatal depression scale (EPDS; Cox et al. 1987). SES was measured with Hollingshead social position criteria including both maternal and paternal education and occupation status with higher scores reflecting higher SES (Hollingshead 1975). In addition, the child's biological sex was controlled for in the analysis.

### 2.3 Statistical Analysis

All analyses were conducted using R software version 4.5.1. Path analysis and multigroup analysis were performed using the *lavaan* package (Rosseel 2012), with maximum likelihood with robust standard errors estimation to account for the non‐normal distributions. For the multigroup analysis, which examined whether the associations varied across trimesters of prenatal stress exposure, model invariance testing was conducted. This involved comparing an unconstrained model, in which path coefficients were allowed to vary across the three trimester groups, to a constrained model, in which coefficients were held equal across trimesters. To determine whether the more constrained model exhibited a substantial decrease in model fit compared to the less constrained model, the change in the comparative fit index (ΔCFI) was assessed, with a threshold of ΔCFI < 0.01 indicating no substantial difference in fit (Hirschfeld and Von Brachel 2014). In cases where significant differences in specific path coefficients were identified, these coefficients were selectively freed to improve model fit and better capture trimester‐specific effects.

To evaluate assumptions regarding missing data, logistic regression analyses were performed, as 88 participants (40.55%) had missing LQ data, primarily due to attrition and incomplete questionnaire return over the course of the longitudinal follow‐up. Missingness was significantly predicted by SES (OR = 0.97, 95% CI [0.95, 0.99]) and gestational age at birth (OR = 0.85, 95% CI [0.73, 1.00]), suggesting that data were likely missing at random. Consequently, Full Information Maximum Likelihood estimation was applied to handle missing data.

The path analysis included the following variables: Child biological sex, SES, gestational age, postnatal maternal depression symptoms (EPDS), measures of objective hardship (IF100), and subjective distress (COSMOSS), with handedness (LQ) as the dependent variable. The predictors and outcome were allowed to covary.

## 3 Results

### 3.1 Descriptive Statistics

Descriptive statistics are presented in Table 1 and the correlation matrix between all the variables is presented in Table 2.

**TABLE 1:** Sample description by trimester of flood exposure.

	Total sample (*n* = 217)					
	Mean (SD)					
	First trimester (*n* = 66)		Second trimester (*n* = 88)		Third trimester (*n* = 63)	
Predictor variables	Females (*n* = 33)	Males (*n* = 32)	Females (*n* = 35)	Males (*n* = 51)	Females (*n* = 31)	Males (*n* = 32)
SES	51.40 (10.80)	51.60 (11.40)	50.30 (11.60)	51.50 (9.46)	52.80 (11.00)	53.20 (8.54)
Gestational age	39.1 (1.36)	39.4 (1.20)	39.1 (1.48)	39.10 (1.41)	39.60 (1.09)	39.30 (1.42)
EPDS	6.79 (5.38)	8.31 (5.73)	6.00 (3.96)	5.48 (4.28)	6.84 (4.35)	6.53 (4.75)
IF100	6.70 (10.10)	8.06 (10.30)	11.10 (13.50)	8.98 (11.8)	8.13 (8.73)	8.97 (8.81)
COSMOSS	−0.18 (0.82)	0.15 (1.01)	0.18 (0.92)	0.05 (0.96)	−0.21 (0.76)	0.16 (0.98)
LQ	91.30 (13.20)	72.6 (34.8)	84.80 (26.80)	79.20 (24.10)	83.60 (25.90)	86.30 (18.90)

*Note:* Data on biological sex were missing for three children.

Abbreviations: COSMOSS, subjective distress; EPDS, postnatal maternal depression symptoms; IF100, objective hardship; LQ, laterality quotient; SES, socioeconomic status.

**TABLE 2:** Correlation matrix.

Variables	SES	Gestational age	EPDS	IF100	COSMOSS
SES	—	—	—	—	—
Gestational age	0.02	—	—	—	—
EPDS	−0.23^***^	−0.04	—	—	—
IF100	−0.24^***^	−0.02	0.18^**^	—	—
COSMOSS	−0.19^**^	0.05	0.36^***^	0.48^***^	—
LQ	0.07	−0.11	−0.14	−0.10	−0.14

Abbreviations: COSMOSS, subjective distress; EPDS, postnatal maternal depression symptoms; IF100, objective hardship; LQ, laterality quotient; SES, socioeconomic status.

* *p* < 0.05

** *p* < 0.01

*** *p* < 0.001.

A series of ANOVAs were conducted to examine differences in the variables based on the trimester of stress exposure. EPDS scores differed significantly across trimesters, F(2, 207) = 3.39, *p* = 0.036, *η_p_* ^2^ = 0.032. Post hoc Tukey's HSD test indicated that individuals exposed to stress during the first trimester (*M* = 7.65, SD = 5.60) had significantly higher scores on the EPDS than those exposed during the second trimester (*M* = 5.63, SD = 4.10), *p* = 0.027, *d* = 0.428. However, the EPDS scores in the first and second trimester groups did not significantly differ from those in the third trimester (*M* = 6.69, SD = 4.52). In contrast, no significant trimester‐related differences were observed for SES (F(2, 214) = 0.68, *p* = 0.510, *η_p_* ^2^ = 0.006), gestation age at birth (F(2, 204) = 1.08, *p* = 0.342, *η_p_* ^2^ = 0.010), IF100 (F(2, 214) = 0.58, *p* = 0.559, *η_p_* ^2^ = 0.005), COSMOSS (F(2, 214) = 0.28, *p* = 0.758, *η_p_* ^2^ = .003), or LQ (F(2, 126) = 0.17, *p* = 0.840, *η_p_* ^2^ = 0.003).

For descriptive purposes, handedness directionality was estimated using two approaches employing different formulas and cut‐offs. This decision was motivated by prior methodological work addressing the operationalization of weak‐handedness, which has led to the use of different computation methods and thresholds (Fagard et al. 2015; Mundorf et al. 2024). First, following Fagard et al. (2015), handedness was calculated using the formula [(R − L) / total number of responses] × 100, with weak handedness defined by values between −30 and +30. Using this approach, 83.8% of children were classified as right‐handed, 11.7% as left‐handed, and 4.5% as weak‐handed. Second, following Mundorf et al. (2024) and Vingerhoets et al. (2023), handedness was computed using the LQ [(R − L) / (R + L)] × 100, with weak handedness defined by values between −60 and +60. This approach yielded highly similar proportions, with 83.1% right‐handed, 12.3% left‐handed, and 4.5% weak‐handed children.

### 3.2 PNMS and Weak Handedness

A multigroup analysis was conducted to test the hypothesis, as it allows for the examination of whether PNMS is associated with child handedness and, if so, whether these associations vary across trimesters of stress exposure, while controlling for key covariates. The multigroup analysis indicated that the constrained model, which imposed equality of path coefficients across trimesters, exhibited a poorer fit than the unconstrained model (ΔCFI > 0.01). Model fit improved when allowing two path coefficients, sex and IF100 predicting LQ, to vary across trimesters. The adjusted constrained model showed better fit indices then the unconstrained model and demonstrated good fit to the data (scaled *χ* ^2^(27) = 40.16, robust CFI = 0.97, robust TLI = 0.95, robust RMSEA = 0.036, SRMR = 0.081).

Sex significantly predicted offspring handedness among mothers exposed to the natural disaster during the first trimester. Specifically, being male was associated with increased weak handedness in first‐trimester (*β* = −0.29, *p* = 0.036). However, no significant association between sex and LQ was observed for mothers exposed to stress during the second or third trimesters (see Table 3 for the full results). As for IF100, it did not predict offspring handedness among mothers exposed to stress during the first or second trimester (see Table 3). In contrast, for third‐trimester exposure, IF100 was significantly associated with LQ, with higher IF100 (i.e., greater objective hardship) being associated with increased weak handedness in children (*β* = −0.34, *p* = 0.015). No other predictors were significant (see Table 3).

**TABLE 3:** Path coefficients predicting the laterality quotient (LQ).

	First trimester (*R* ^2^ = 0.16)					Second trimester (*R* ^2^ = 0.07)					Third trimester (*R* ^2^ = 0.21)				
Variables	*B*	SE	CI	*β*	*p*	*b*	SE	CI	*β*	*p*	*b*	SE	CI	*β*	*p*
Child's sex (Male)	**−15.94**	**7.60**	**−30.85** to **−1.03**	**−0.30**	**0.036**	−7.84	7.24	−22.02–6.35	−0.16	0.279	5.11	6.54	−7.72–17.93	0.11	0.435
SES	0.08	0.18	−0.27–0.44	0.03	0.641	0.08	0.18	−0.27–0.44	0.03	0.641	0.08	0.18	−0.27–0.44	0.03	0.641
Gestational age	−2.36	1.39	−5.08–0.37	−0.11	0.09	−2.36	1.39	−5.08–0.37	−0.11	0.09	−2.36	1.39	−5.08–0.37	−0.11	0.09
EPDS	−0.70	0.54	−1.76–0.37	−0.14	0.202	−0.70	0.54	−1.76–0.37	−0.14	0.202	−0.70	0.54	−1.76–0.37	−0.14	0.202
IF100	−0.22	0.58	−1.35–0.91	−0.09	0.701	0.25	0.33	−0.389–0.89	0.13	0.439	**−0.90**	**0.37**	**−1.63 to −0.18**	**−0.34**	**0.015**
COSMOSS	−2.47	3.63	−9.58–4.64	−0.09	0.495	−2.47	3.63	−9.58–4.64	−0.09	0.495	−2.47	3.63	−9.58–4.64	−0.09	0.495

*Note:* Only the coefficients of maternal handedness and IF100 are allowed to vary; Child's sex was coded as 0 = female, 1 = male; Bold: *p*‐values indicating significance (*p* < 0.05).

Abbreviations: CI, b's 95% confidence interval; COSMOSS, subjective distress; EPDS, postnatal maternal depression symptoms; IF100, objective hardship; LQ, laterality quotient; SE, standard error.

Given the correlation between IF100 and COSMOSS (see Table 2), sensitivity analyses were conducted to evaluate potential multicollinearity and assess the robustness of the findings across different model specifications. Specifically, we examined whether the observed associations persisted when these predictors were included separately in the model. When COSMOSS was excluded from the model, IF100 remained non‐significant for first‐ and second‐trimester exposure (*β* = −0.12, *p* = 0.614; *β* = 0.08, *p* = 0.590, respectively) but reached significance for third‐trimester exposure (*β* = −0.35, *p* = 0.009). Conversely, when IF100 was excluded, COSMOSS remained non‐significant across all trimesters (first trimester: *β* = 0.07, *p* = 0.668; second trimester: *β* = −0.23, *p* = 0.234; third trimester: *β* = −0.09, *p* = 0.701).

We conducted additional sensitivity analyses to evaluate the robustness of our findings. Following the approach of Glover et al. (2004), who examined hand preference as both a continuous and a categorical variable, we transformed our continuous LQ into a categorical variable (see Supporting Information 1 for a detailed description of the procedure). Logistic regression revealed a significant interaction between sex and trimester, indicating that the odds of weak‐handedness varied across trimesters for males and females (OR = 0.04, 95% CI [0.003, 0.59], *p* = 0.019; see Table S1 in Supporting Information 1). Specifically, in the first trimester, males had a substantially higher probability of being weak‐handed (35.4%) compared to females (5.3%), whereas sex differences were smaller in the second and third trimesters (see Table S2 in Supporting Information 1). No other significant predictors or interactions were found (see Table S1 in Supporting Information 1).

Although the effect of objective prenatal stress (IF100) did not reach statistical significance in this sensitivity analysis, we examined the estimated probabilities since this predictor was significant in the original analyses (when using a continuous LQ). The estimated probabilities of weak‐handedness followed a trimester‐specific pattern. In the first trimester, probabilities increased modestly from 9.7% at −1 SD to 22.1% at +1 SD; in the second trimester, probabilities remained around 25%; and in the third trimester, probabilities increased substantially from 12.5% at −1 SD to 38.0% at +1 SD (see Table S3 in Supporting Information 1). Overall, these results are consistent with our original analyses using continuous LQ. Full model coefficients of this sensitivity analysis, including all main effects, interaction terms, and estimated probabilities, are provided in Tables S1‐S5 in Supporting Information 1.

## 4 Discussion

The present study aimed to examine the extent to which in utero exposure to objective hardship and subjective distress, as well as the gestational timing of this exposure, are associated with an increased prevalence of weak handedness in offspring at 60 months of age, as reported by their mothers. By analyzing data from the Iowa Flood Study cohort, we were able to investigate rare data of the longitudinal effects of PNMS from a natural experiment on handedness development in children. After controlling for child sex, birth‐related stressors (i.e., gestational age at birth), and postnatal maternal depression, the findings revealed that sex was a significant predictor of child handedness, but only when the natural disaster occurred during the first trimester. In contrast, objective stress exposure (e.g., threat and loss) during the third trimester emerged as a significant predictor of increased weak handedness in children, whereas no such effect was observed for earlier trimesters. Finally, subjective stress exposure (e.g., PTSD symptoms) was not significantly associated with children's handedness outcomes. These findings highlight the critical role of prenatal stress timing in shaping the development of handedness, with distinct associations for child sex and objective hardship, but not for subjective maternal distress.

The results partially support our hypothesis, demonstrating an association between weak handedness in children and maternal exposure to objective hardship, but not to subjective maternal distress. This aligns with prior research reporting associations between objective maternal stressors and offspring weak handedness (Gutteling et al. 2007; Obel et al. 2003; Rodriguez and Waldenström 2008), yet contrast with other results suggesting that prenatal subjective distress, such as maternal well‐being, are associated with child weak handedness (Gutteling et al. 2007; Obel et al. 2003; Rodriguez and Waldenström 2008). Although the association between postnatal maternal depression, assessed by the EPDS, and weak‐handedness was not statistically significant in our sample, the non‐trivial effect size (*β* = −14) suggests that this association may be non‐significant due to limited statistical power arising from the small sample size. Notably, previous research with a larger sample (*n* = 1714) has reported a significant association between maternal depression assessed by the EPDS and increased weak handedness in offspring (Rodriguez and Waldenström 2008).

The observed differences between the effects of objective and subjective maternal stress in this study may be linked to epigenetic mechanisms. A longitudinal study investigating the effects of a natural disaster (Project Ice Storm) found that objective maternal stress was associated with alterations in offspring DNA methylation, while subjective distress was not (Cao‐Lei et al. 2014). These findings suggest that objective stress might induce epigenetic changes that influence neurodevelopmental outcomes. Handedness is known to be partly heritable and influenced by genetic factors (Cuellar‐Partida et al. 2021; McManus 2022). In this study, the observed sex differences in weak handedness during the first trimester may reflect genetic influences, with males being more likely to exhibit increased weak handedness as in the general population. Previous research suggests that these sex differences could be partly mediated by genetic factors, such as the X chromosome and variations in androgen receptor length (Arning et al. 2015; Papadatou‐Pastou et al. 2020). However, when objective stress emerged as a predictor of weak handedness, sex differences ceased to be a significant predictor, implying that environmental factors, likely mediated by epigenetic modifications, may disrupt typical patterns of heritability in lateralization. This hypothesis aligns with perspectives on epigenetic mechanisms in lateralization, which suggest that environmental influences, such as maternal prenatal stress, can induce epigenetic modifications that affect protein synthesis and brain development, including the development of cerebral lateralization and handedness (Schmitz et al. 2017).

One possible neurobiological explanation regarding the association between PNMS (i.e., objective stress) and handedness involves the impact of maternal stress exposure on fetal brain development via hormonal mechanisms. Prenatal stress can dysregulate the hypothalamic‐pituitary‐adrenal (HPA) axis, leading to elevated cortisol levels that may disrupt neurodevelopmental processes (Glover et al. 2010). Early alterations in the HPA axis function may be implicated in the development of atypical brain lateralization, which may contribute to a higher prevalence of weak handedness (Berretz et al. 2020; Hamaoui et al. 2024). In addition, stress‐induced disruptions in interhemispheric connectivity may account for the observed increase in weak handedness, as some authors suggest that PNMS could affect corpus callosum development (Charil et al. 2010; Coe et al. 2002; Rodriguez and Waldenström 2008), which could in turn influence cerebral lateralization (Ocklenburg et al. 2016).

The gestational timing of prenatal stress exposure emerged as a critical factor. The results partially supported our hypothesis, proposing that only stress exposure during the third trimester was associated with weak handedness in children. This finding aligns with previous research by Obel et al. (2003) and Gutteling et al. (2007), which reported stronger associations between maternal stress in late pregnancy and weak handedness. These results are consistent with the fetal programming theory, which suggests that prenatal vulnerabilities vary across different gestational stages. As various brain systems mature at distinct points during fetal development, environmental adversities during sensitive developmental windows can induce lasting, yet distinct, neurodevelopmental changes (Glover et al. 2010; Simcock et al. 2016). The present findings are consistent with evidence linking third‐trimester PNMS to alterations in the infant's motor system. Studies on natural disasters have demonstrated that stress exposure in the later stages of pregnancy is associated with poorer motor development (Simcock et al. 2016), including deficits in visual‐motor integration (Cao et al. 2014). Therefore, it is plausible that the in utero maturation of motor system underlying lateralized fetal motor behaviors and subsequent handedness (Parma et al. 2017) is particularly vulnerable during the third trimester of pregnancy.

This study has several limitations. The small sample size may have resulted in insufficient statistical power, potentially affecting the ability to detect smaller but meaningful associations (e.g., between maternal depression and offspring handedness). In addition, the present study did not include direct measures of hand performance, which would have allowed a more precise assessment of ambidexterity, in line with recommendations from the LICI. Future studies should ideally assess ambidexterity using performance‐based measures rather than relying solely on hand preference questionnaires (Vingerhoets et al. 2023). A related limitation is that mixed‐handedness and ambidexterity were not analyzed as separate categories. LQs can reflect either inconsistent hand use across tasks or true ambidexterity, two distinct constructs that are frequently conflated when inferred from preference‐based indices alone (Packheiser et al. 2025). Distinguishing these two forms of weak handedness could yield more nuanced insights in examining associations between weak handedness and neurodevelopmental or psychiatric disorders. Handedness was assessed through maternal report, which may introduce bias, as maternal perceptions may not always align with objective measures. Furthermore, the absence of parental handedness data limits the ability to adequately account for genetic influences on handedness. Limitations also arise in the control of gestational age at birth. Prior research indicates that very preterm (<32 weeks gestation) and extremely preterm (<28 weeks gestation) births are associated with atypical handedness (Hamaoui et al. 2023). However, in this sample, only two children were born before 32 weeks of gestation, limiting the ability to disentangle the effects of PNMS from those of birth‐related stressors such as prematurity. Finally, the study sample consisted exclusively of mothers exposed to a natural disaster, without a comparison group of mothers with typical pregnancies. This restricts the generalizability of the findings. However, the sample exhibited substantial variability in stress exposure levels. For instance, with regard to objective stress, some mothers reported no exposure (IF100 = 0, *n* = 23, 8.6%), whereas others experienced much higher levels (IF100 = 50, *n* = 5, 1.9%). This good variability in stress exposure reduces the limitations in our sample associated with studying only disaster‐exposed pregnancies, allowing for some degree of generalizability of the results to other, more common stressors that have both objective and subjective aspects, such as bereavement.

Despite these limitations, the study also possesses several notable strengths. First, children's handedness was assessed at age 5, just prior to school entry, a period during which hand preference reflects several years of postnatal organization and consolidation, while remaining relatively less exposed to formal schooling‐related social pressures. Assessing handedness at this time point enables the examination of how early environmental factors contribute to the developmental trajectory of handedness before the increasing influence of explicit educational and cultural constraints. Second, the study controlled for important confounding variables (e.g., birth‐related stressors, child sex, postnatal maternal depression), strengthening the internal validity of the findings by isolating the specific effects of prenatal stress on handedness development. Third, the study's focus on prenatal stress induced by a sudden‐onset natural disaster enhances its internal and ecological validity, and the precision of the gestational timing of exposure measure, providing insights into the real‐world impact of environmental stressors on child neurodevelopment. In addition, handedness was measured using a clearly defined, theoretically informed approach, applying an LQ derived from a validated questionnaire, which captures different categories of hand preference and aligns with recent evidence linking it to hand performance tasks. Comprehensive sensitivity analyses were conducted, examining both continuous and categorical definitions of handedness and systematically testing the robustness of results across different covariate specifications. The analytic procedure employed path analyses with full handling of missing data and multigroup modeling, enabling robust estimation of associations while accounting for developmental timing and relevant covariates. Finally, the longitudinal design strengthens the evidence for potential causal effects of prenatal stress on handedness development. Together, these methodological and conceptual features provide an ecologically grounded and rigorous evaluation of the associations between prenatal stress and weak‐handedness in early childhood.

Future research should investigate the underlying neurobiological mechanisms of these effects, particularly examining fetal cortisol exposure and alterations in neural connectivity. Furthermore, while behavioral lateralization, such as handedness, serves as an indirect, though imperfect, indicator of cerebral lateralization (Ocklenburg et al. 2024), additional measures of laterality, such as brain imaging, are essential for gaining a more comprehensive understanding of these processes. In addition, examining long‐term cognitive and motor outcomes in children with weak handedness could clarify whether PNMS‐induced changes in lateralization have implications for later developmental trajectories. The present analyses did not include information on formal child neurodevelopmental diagnoses, which is relevant given prior evidence‐linking atypical handedness to neurodevelopmental disorders. Future studies incorporating standardized or parent‐reported diagnostic measures will be important to clarify whether associations between handedness and psychopathology reflect independent developmental pathways or partially overlapping mechanisms.

Finally, future research would benefit from adopting a developmental perspective on handedness by examining its trajectory across childhood, rather than relying on single time‐point assessments. Such an approach would allow for an evaluation of the hypothesis that prenatal stress may be associated with a delay in the consolidation of handedness (Gutteling et al. 2007). This hypothesis was proposed to account for inconsistencies across studies conducted at different ages, whereby associations between PNMS and mixed‐handedness have been observed in early childhood in some studies, but only in later childhood in others, depending on the timing of stress exposure during gestation. According to this hypothesis, the association between PNMS and weak‐handedness may be influenced both by the gestational timing of stress and by the developmental period at which handedness is assessed. Exposure to prenatal stress earlier in gestation may slow the consolidation of hand preference, producing effects that are most evident in early childhood and potentially diminishing with age, as weak‐handedness is more prevalent in early childhood and hand preference typically stabilizes over development. In contrast, exposure later in pregnancy may alter the developmental trajectory of handedness in ways that remain detectable beyond early childhood. Longitudinal studies incorporating repeated assessments of handedness from early childhood onward, ideally combining preference‐based indicators with performance‐based measures, will therefore be necessary to clarify how prenatal stress relates to the developmental trajectory of handedness.

## 5 Conclusion

This study underscores the trimester‐dependent effects of sex and PNMS on offspring handedness, highlighting the role of late gestational objective stress in shaping weak handedness, while sex exerts influence when stress occurs during the first trimester. From a theoretical standpoint, these findings support the hypothesis that handedness ontogenesis begins in utero (Michel 2021) and is also shaped by genetic factors (Cuellar‐Partida et al. 2021; McManus 2022). However, epigenetic mechanisms may disrupt this genetic pathway, and environmental factors could further modulate the developmental trajectory of laterality (Schmitz et al. 2017). These results also have clinical implications. Given the established associations between non‐right‐handedness (e.g., mixed‐handedness) and various neurodevelopmental and psychiatric disorders (Borawski et al. 2023; Hirnstein and Hugdahl 2014; Markou et al. 2017; Packheiser et al. 2023; Somers et al. 2009), weak handedness may serve as a potential biomarker for such disorders, reflecting the long‐term effects of prenatal stress on neurodevelopmental trajectories (Ocklenburg et al. 2024). Nonetheless, further investigation is necessary to better understand this association and its underlying mechanisms.

## Funding

This study was funded by grants from the Canadian Institutes of Health Research (MOP 93660) and the National Institute of Mental Health—RAPID grant (1R21MH086150) to S.K. and Michael W. O'Hara, and additional funding awarded to J.H. through the Research Network on the Developmental and Intergenerational Origins of Children's Health (ODISE network).

## Conflicts of Interest

The authors declare no conflicts of interest.

## Supporting information




**Supplementary Materials**: dev70143‐sup‐0001‐SuppMat.docx

## Data Availability

The data that support the findings of this study are available on request from the corresponding author, S.K.
