# Supplementary material for: Prenatal Maternal Stress and Weak Handedness in Early Childhood: The Iowa Flood Study
Source: Dev Psychobiol. 2026 Mar 17;68(2):e70143. doi: 10.1002/dev.70143 (PMC12995510; doi:10.1002/dev.70143)
Supplement: Supplementary file 1 — Supplementary Materials: dev70143‐sup‐0001‐SuppMat.docx [file DEV-68-e70143-s001.docx]

**Supplementary material 1 – Hand preference as a categorical variable**

We conducted sensitivity analyses to assess the robustness of our findings. Following Glover et al. (2004), who analyzed the laterality quotient (LQ) as both a continuous and categorical variable, we transformed our continuous LQ into a categorical measure. However, our relatively small sample size (n = 217) posed challenges for categorical analyses. Specifically, applying the LQ formula ∣(R − L) / total responses × 100∣ with a cut-off of <30 for weak-handedness, while including predictors such as trimester and sex, resulted in very low cell counts in the weak-handed category, rendering logistic regression estimates unreliable.

We therefore conducted exploratory analyses using progressively less conservative cut-offs. A threshold of <40 did not sufficiently increase cell counts, whereas a cut-off of <60 (corresponding to children reporting “either hand” on at least 2 items) produced a distribution of 22% weak-handed and 78% strong-handed children, allowing logistic regression models to converge. Accordingly, a logistic regression was conducted to examine predictors of weak-handedness at 60 months. A significant interaction was observed between sex and trimester, indicating that the odds of weak-handedness differed for males and females across trimesters (see Table S1).

Table S1. Logisitic regression with handedness categorized as weak vs strong handers

| Predictor | Estimate | OR | 95% CI | *p*-value |
| --- | --- | --- | --- | --- |
| **Sex (male vs female)** | **2.29** | **9.85** | **1.25 – 77.44** | **.030** |
| IF100 | 0.06 | 1.06 | 0.95 – 1.18 | .292 |
| Trimester (2nd vs 1st) | −4.29 | 0.01 | 0 – 15.10 | .230 |
| Trimester (3rd vs 1st) | −0.75 | 0.47 | 0 – 468.94 | .831 |
| EDPS | −0.01 | 0.99 | 0.83 – 1.18 | .915 |
| Cosmoss | −0.20 | 0.82 | 0.27 – 2.47 | .720 |
| Gestational age | 0.32 | 1.37 | 0.93 – 2.03 | .110 |
| SES | −0.08 | 0.92 | 0.84 – 1.01 | .074 |
| IF100 × Trimester (2nd vs 1st) | −0.06 | 0.94 | 0.82 – 1.09 | .420 |
| IF100 × Trimester (3rd vs 1st) | 0.03 | 1.03 | 0.88 – 1.21 | .722 |
| Sex × Trimester (male × 2nd vs female × 1st) | −1.45 | 0.24 | 0.02 – 3.11 | .272 |
| **Sex × Trimester (male × 3rd vs female × 1st)** | **−3.20** | **0.04** | **0.003 – 0.59** | **.019** |
| Cosmoss × Trimester (2nd vs 1st) | 0.71 | 2.04 | 0.41 – 10.13 | .385 |
| Cosmoss × Trimester (3rd vs 1st) | −0.25 | 0.78 | 0.13 – 4.58 | .782 |
| EDPS × Trimester (2nd vs 1st) | 0.18 | 1.20 | 0.90 – 1.59 | .214 |
| EDPS × Trimester (3rd vs 1st) | −0.02 | 0.98 | 0.74 – 1.30 | .873 |
| SES × Trimester (2nd vs 1st) | 0.10 | 1.10 | 0.97 – 1.24 | .129 |
| SES × Trimester (3rd vs 1st) | 0.05 | 1.05 | 0.94 – 1.19 | .381 |

Note. Estimates represent the log adds of “LQ = Weak” vs. “LQ = Strong”. Bold: p-values indicating significance (p < .05); CI: 95% confidence interval; EPDS: Postnatal maternal depression symptoms; IF100: Objective hardship; COSMOSS: Subjective distress.

The probability of weak-handedness varied across sex and trimester of prenatal exposure (see Table S2). In the first trimester, males showed a markedly higher probability of being weak-handed (35.4%) compared to females (5.3%). In the second and third trimester, probabilities were more similar between males and females (see Table S2).

Table S2. Estimated probability of weak-handedness by sex and trimester

| Trimester | Sex | Probability | SE | 95% CI (Lower – Upper) |
| --- | --- | --- | --- | --- |
| First | Female | 0.053 | 0.046 | 0.009 – 0.256 |
| First | Male | 0.354 | 0.131 | 0.152 – 0.627 |
| Second | Female | 0.180 | 0.094 | 0.059 – 0.434 |
| Second | Male | 0.337 | 0.113 | 0.159 – 0.577 |
| Third | Female | 0.318 | 0.118 | 0.139 – 0.575 |
| Third | Male | 0.157 | 0.090 | 0.047 – 0.415 |

Note: Probabilities represent the estimated likelihood of being classified as weak-handed based on sex and trimester of prenatal stress exposure.

Although the effect did not reach statistical significance in the categorical analyses, we examined the estimated probabilities associated with objective prenatal stress because this predictor was significant in the original analyses using continuous LQ. The probability of weak-handedness increased with higher levels of objective prenatal stress (IF100) in a trimester-specific manner (Table S3). In the first trimester, probabilities rose modestly from 9.7% at −1 SD to 22.1% at +1 SD of IF100. In the second trimester, probabilities were consistently around 25% across stress levels. In contrast, in the third trimester, the probability of weak-handedness increased substantially with higher stress, from 12.5% at −1 SD to 38.0% at +1 SD (see Table S3).

Table S3. Estimated probability of weak-handedness by objective stress and trimester

| Trimester | IF100 (SD) | Probability | SE | 95% CI (Lower – Upper) |
| --- | --- | --- | --- | --- |
| First | −1 SD | 0.097 | 0.071 | 0.022 – 0.344 |
| First | Mean | 0.149 | 0.071 | 0.055 – 0.346 |
| First | +1 SD | 0.221 | 0.110 | 0.075 – 0.497 |
| Second | −1 SD | 0.249 | 0.099 | 0.106 – 0.483 |
| Second | Mean | 0.250 | 0.078 | 0.129 – 0.430 |
| Second | +1 SD | 0.252 | 0.111 | 0.096 – 0.516 |
| Third | −1 SD | 0.125 | 0.078 | 0.034 – 0.365 |
| Third | Mean | 0.228 | 0.077 | 0.111 – 0.411 |
| Third | +1 SD | 0.380 | 0.147 | 0.153 – 0.675 |

Note: Probabilities represent the estimated likelihood of being classified as weak-handed based on objective stress and trimester of prenatal stress exposure.

For maternal depressive symptoms and subjective prenatal stress, neither the continuous nor the categorical LQ analyses yielded statistically significant effects. We nonetheless report the estimated probabilities below to provide a complete account of the results.

For maternal depressive symptoms (EDPS), in the first trimester, probabilities were relatively sTable S1cross depression levels (14–15%). In the second trimester, higher depressive symptoms were associated with an increased probability of weak-handedness, from 13.2% at −1 SD to 42.4% at +1 SD. In the third trimester, probabilities showed moderate changes (20–26%) across depression levels (see Table S4).

Table S4. Estimated probability of weak-handedness by depression and trimester

| Trimester | EDPS (SD) | Probability | SE | 95% CI (Lower – Upper) |
| --- | --- | --- | --- | --- |
| First | −1 SD | 0.154 | 0.102 | 0.038 – 0.457 |
| First | Mean | 0.149 | 0.071 | 0.055 – 0.346 |
| First | +1 SD | 0.143 | 0.075 | 0.048 – 0.355 |
| Second | −1 SD | 0.132 | 0.067 | 0.046 – 0.325 |
| Second | Mean | 0.250 | 0.078 | 0.129 – 0.430 |
| Second | +1 SD | 0.424 | 0.180 | 0.147 – 0.758 |
| Third | −1 SD | 0.256 | 0.135 | 0.079 – 0.578 |
| Third | Mean | 0.228 | 0.077 | 0.111 – 0.411 |
| Third | +1 SD | 0.203 | 0.109 | 0.063 – 0.489 |

Note: Probabilities represent the estimated likelihood of weak-handedness across levels of maternal depressive symptoms in each trimester.

For the subjective prenatal stress (COSMOSS), in the second trimester, probability increased with higher stress, from 18.0% at −1 SD to 33.7% at +1 SD (see Table S5).

Table S5. Estimated probability of weak-handedness by subjective stress and trimester

| Trimester | COSMOSS (SD) | Probability |  | SE | 95% CI (Lower – Upper) |
| --- | --- | --- | --- | --- | --- |
| First | −1 SD | 0.171 |  | 0.100 | 0.049 – 0.451 |
| First | Mean | 0.149 |  | 0.071 | 0.055 – 0.346 |
| First | +1 SD | 0.129 |  | 0.085 | 0.032 – 0.396 |
| Second | −1 SD | 0.180 |  | 0.109 | 0.049 – 0.484 |
| Second | Mean | 0.250 |  | 0.078 | 0.129 – 0.430 |
| Second | +1 SD | 0.337 |  | 0.118 | 0.153 – 0.590 |
| Third | −1 SD | 0.301 |  | 0.120 | 0.123 – 0.569 |
| Third | Mean | 0.228 |  | 0.077 | 0.111 – 0.411 |
| Third | +1 SD | 0.169 |  | 0.121 | 0.036 – 0.523 |

Note: Probabilities represent the estimated likelihood of weak-handedness across levels of maternal subjective stress in each trimester.
